# Multiscale Characterization of Type I Collagen Fibril Stress–Strain Behavior under Tensile Load: Analytical vs. MD Approaches

**DOI:** 10.3390/bioengineering9050193

**Published:** 2022-04-28

**Authors:** Afif Gouissem, Raouf Mbarki, Fadi Al Khatib, Malek Adouni

**Affiliations:** 1 Mechanical Engineering Department, Australian University, East Mishref, Kuwait City P.O. Box 1411, Kuwait; a.gouissem@ack.edu.kw (A.G.); r.mbrki@ack.edu.kw (R.M.); f.akhatib@ack.edu.kw (F.A.K.); 2 Physical Medicine and Rehabilitation Department, Northwestern University, Chicago, IL 60611, USA

**Keywords:** analytical formulation, coarse-grained model, collagen, crosslinks, fibril, stress–strain curve, molecular dynamics

## Abstract

Type I collagen is one of the most important proteins in the human body because of its role in providing structural support to the extracellular matrix of the connective tissues. Understanding its mechanical properties was widely investigated using experimental testing as well as molecular and finite element simulations. In this work, we present a new approach for defining the properties of the type I collagen fibrils by analytically formulating its response when subjected to a tensile load and investigating the effects of enzymatic crosslinks on the behavioral response. We reveal some of the shortcomings of the molecular dynamics (MD) method and how they affect the obtained stress–strain behavior of the fibril, and we prove that not only does MD underestimate the Young’s modulus and the ultimate tensile strength of the collagen fibrils, but also fails to detect the mechanics of some stretching phases of the fibril. We prove that non-crosslinked fibrils have three tension phases: (i) an initial elastic deformation corresponding to the collagen molecule uncoiling, (ii) a linear regime related to the stretching of the backbone of the tropocollagen molecules, and (iii) a plastic regime dominated by molecular sliding. We also show that for crosslinked fibrils, the second regime can be subdivided into three sub-regimes, and we define the properties of each regime. We also prove, analytically, the alleged MD quadratic relation between the ultimate tensile strength of the fibril and the concentration of enzymatic crosslinks (*β*).

## 1. Introduction

Collagen is the major constitutive component of the human connective tissue (~30% of the total protein mass) and is present in most body organs. Currently, more than 20 types of collagen have been identified [1], while other types are still being investigated by means of molecular biology, gene cloning, and other methods.

Among the whole collagen family, types I, II, III, IV, and V are the most abundant: While type I is present in bones and tendons [2], type II widely exists in the cartilage and bones [3], type III is the main constituent of hollow organs such as large blood vessels [4], type VI is widely present in the skin [5], and type V is the prime protein in the corneal stroma and skeletal muscles [6]. Although present in lower proportions, the remaining types of collagens do play a very important role in the human anatomy because of their presence in critical body organs such as the heart, liver, and brain. In particular, type I is the protein attracting most of the scientific attention because of its abundance in critical human parts (represents over 90% of the total collagen content).

Understanding the mechanical behavior and the chemical characterization of this protein has been, and still is, a hot topic in the biomedical field [7,8,9]. This is due to the interlocking relationship between the mechanical properties of the fibrils and some disease-related processes. For example, nonenzymatic crosslinks (AGEs), which are clinically related to a high blood sugar content, can be mechanically linked to the stiffening of the collagen fibrils. Understanding the mechanical effect of the AGEs can provide insights into the chemical processes involved and enables a better clinical understanding of the matter [10,11,12,13]. In addition, other processes occurring in the collagen fibril, such as degradation [14,15], glycation [16,17], or crosslinking [18,19], might be better understood through a mechanical characterization of the impacted fibril. In this context, this work focuses on understanding the mechanical behavior of type I collagen, and the effect of the enzymatic crosslinking on the fibril.

In previous studies, different approaches were used to estimate the response of the collagen fibril under a tensile load: experimental tests have been performed to extract stress–strain curves for tendons, bones, and other organs [20,21,22,23]. Unfortunately, such studies were performed on macro-scale samples including the collagen fibrils and a non-collagenous soft matrix consisting of proteoglycan, water, and other proteins [24], which rendered the extraction of the collagen properties less accurate. Molecular dynamic simulations, on the other hand, target the properties of the fibril itself without the need to pass through the macro-properties of the tissue [25,26]. This method, however, has two major shortcomings. First, due to computational limitations, MD cannot mimic the correct size of the actual collagen fibrils present in the human body. In fact, most MD-related studies are limited to fibrils 20 nm to 25 nm in diameter [18,27,28,29], whereas actual collagen fibrils can reach up to 500 nm in diameter [1]. Second, MD cannot reach a reasonably low tensile strain rate. Strain rates used in molecular dynamic simulations are in the order of $10^{6}\;s^{-1}$ to $10^{8}{\;s}^{-1}$, which is at best five orders of magnitude higher than the actual strain rate fibrils are subjected to during mechanical testing. Such shortcomings (for both approaches) might have a negative effect on the accuracy of the extracted mechanical properties of the collagen fibrils.

This work aims to tackle this problem from a different perspective and to characterize the behavior of fibrils using an analytical approach. We do not claim that this analytical formulation is presented to correct previous MD studies, since it emerges from an empirically defined interatomic potential. However, we ensure that this approach circumvents MD’s two abovementioned weaknesses (namely, the timescale and the size scale). Such relationships may serve as a tool for designing new links between the molecular collagen features and the tissue level materials properties, using the analytical model as a building block. This can also help to answer some still unclear questions about the collagen structure via experimental model parameter optimization.

## 2. Methodology

In order to understand the stress–strain mechanics of the fibril, and quantify the effect of crosslinks, the following steps were taken: first, the stress–strain behavior of a single tropocollagen molecule was mathematically modeled based on the coarse grain model developed in [30]. Then, using the structural geometry of the fibril [31], the stress–strain of the collagen fibril was deduced. Once the mechanical behavior of the non-crosslinked fibril was formulated, the enzymatic crosslinks were added to the structure, and their effects were quantified.

### 2.1. Stress–Strain Curve for the Tropocollagen Molecule

The tropocollagen molecule has a triple helical protein structure consisting of three amino acid chains. It has a diameter of about 1.5 nm and a length of about 300 nm (Protein Data Bank entry 3HR2 experimentally measured by X-ray crystallography) [32].

On a molecular level, MD can simulate the mechanics of the molecule, since the number of atoms is within its computational capabilities (~3134 atoms). However, the coarse-grained mesoscopic approach consists of abbreviating each set of atoms into a single bead (or super-atom) to reduce the computational cost of the simulation. For this mesoscopic approach, we used tropocollagen molecules of 218 beads where the coordinates of the beads were obtained by averaging the positions of adjacent atoms, resulting in a chain structure of quasi-equidistant beads with equilibrium angles ranging from 164° to 180°. Details on the molecular, mesoscopic model parameters were given in [30], and the molecule’s properties are detailed in Table 1.

If the sole purpose of molecular dynamic simulations was to determine the molecule properties, the coarse-grained approach would have been deemed unnecessary, given that the conventional MD is able to provide the needed results. However, since the molecules are the constitutive element of the fibrils, using this approach on a molecular level allowed us to access the fibrillar level inaccessible otherwise by conventional MD. Following the abovementioned mesoscale formulation [30], the potential energy of the molecule is given by:

(1) $$E=E_{bond}+E_{angle}$$

where the bond energy is a hyper-elastic interaction between each two adjacent beads. It is represented by the 3-regime potential energy and is given by:

(2) $$E_{bond}=\{\begin{array}{c} \frac{K_{T0}}{2}{\left(r-r_{0}\right)}^{2}\;\;\;\;\;\;\;\;\;\;\;\;\;\;\;\;\;\;\;\;\;,\;r<r_{1}\\ \frac{K_{T1}}{2}\left(r-\bar{r_{1}}\right)+C_{1}\;\;\;\;\;\;\;\;\;\;\;,\;r_{1}<r<r_{b}\\ C_{2}\;\;\;\;\;\;\;\;\;\;\;\;\;\;\;\;\;\;\;\;\;\;\;\;\;\;\;\;\;\;\;\;\;\;\;\;\;\;\;\;,\;r>r_{b} \end{array}$$

(3) $$F_{bond}=\frac{\partial E_{bond}}{\partial r}=\{\begin{array}{c} K_{T0}\left(r-r_{0}\right)\;\;\;\;\;\;\;\;\;\;\;\;\;\;\;\;\;\;\;\;\;,\;r<r_{1}\\ K_{T1}\left(r-\bar{r_{1}}\right)\;\;\;\;\;\;\;\;\;\;\;,\;r_{1}<r<r_{b}\\ 0\;\;\;\;\;\;\;\;\;\;\;\;\;\;\;\;\;\;\;\;\;\;\;\;\;\;\;\;\;\;\;\;\;\;\;\;\;\;\;\;,\;r>r_{b} \end{array}$$

where $K_{T0}$ and $K_{T1}$ are spring constants, $r_{1}$ is the distance at which the hyper-elastic behavior of the bond is triggered, $r_{b}$ is the bond-breaking distance, and $\bar{r_{1}}$ and $C_{1}$ are constants calculated to ensure the continuity of the force field and are given by:

(4) $$\bar{r_{1}}=r_{1}-\frac{K_{T0}}{K_{T1}}\left(r_{1}-r_{0}\right)$$

(5) $$C_{1}=\frac{K_{T0}}{2}{\left(r_{1}-r_{0}\right)}^{2}-\frac{K_{T1}}{2}{\left(r_{1}-\bar{r_{1}}\right)}^{2\;}$$

The angular energy $E_{angle}$ is a harmonic three-body interaction between each three adjacent super atoms to control the bending angle between the beads.

(6) $$E_{angle}=K_{\theta }{\left(\theta -\theta_{0}\right)}^{2}\;$$

where $K_{\theta }$ represents the bending strength, $\theta_{0}$ represents the equilibrium angle, and θ represents the actual angle between the three consecutive beads. Parameters for bond and angular energy are given in Table 2.

**Figure 1:**
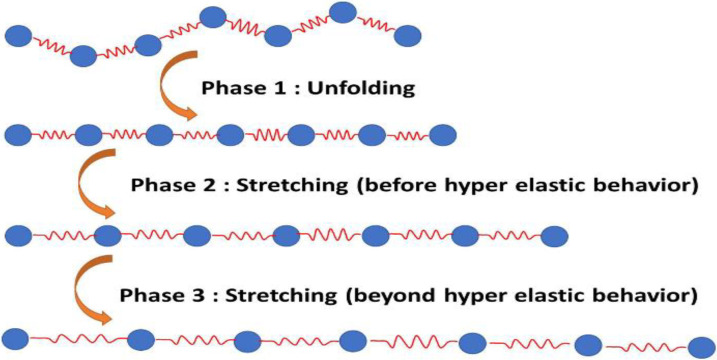
Tropocollagen molecule tensile phases.

Based on the potential forcefield described above, the molecule’s behavior can be summarized in three stages: the unfolding of the molecule, the stretching before the hyperelastic behavior, and the stretching after the hyperelastic behavior (Figure 1).

#### 2.1.1. Stage 1: Unfolding of the Molecule

Based on the mesoscopic parameters presented in Table 2, the straightening of the molecule requires very little energy compared to the energy needed to stretch the bonds. For example, when three consecutive beads have a misalignment of 10° (median misalignment along the molecule), the potential energy needed to straighten the beads is ~0.45 Kcal/mol; the same energy is only able to stretch a single bond by ~0.026 Å. Therefore, very little bond stretching will occur before all beads of the molecules are aligned, which justifies the assumption that the unfolding and the stretching can be uncoupled and hence the stretching phase can be assumed to start only when the unfolding of the molecule is complete.

Once the tensile force is applied to the molecule, the angles between the beads will converge to θ=180°. At this stage, the force between the beads will not be uniform throughout the molecule because the equilibrium angles are different. The maximum force will be at the locations with the highest misalignment (with the furthest equilibrium angle from 180°). The stress and strain at the issue of this stage are denoted as $\varepsilon_{1}^{M}$ and $\sigma_{1}^{M}$, respectively, where the subscript “1” indicates the first phase, and the superscript “*M*” indicates that the property is related to the molecule.

(7) $$\{\begin{array}{c} \varepsilon_{1}^{M}=\frac{n\times r_{0}}{L_{0}}-1\;\sim \;1.36\%\\ F_{1}^{M}=-2K_{\theta }\;\left(\theta_{max}-\theta_{0}\right)\sim \;2.08\times 10^{-11}\;N\\ \sigma_{1}^{M}=\frac{F_{1}^{M}}{A_{mol}}\;\sim \;9.68\;\mathrm{Mpa} \end{array}$$

The Young modulus in this stage is denoted as $E_{1}^{M}$, and is given by:

(8) $$E_{1}^{M}=\frac{\sigma_{1}^{M}}{\varepsilon_{1}^{M}}\;\sim \;711\;\mathrm{Mpa}$$

where *n* and *L*_0_ represent, respectively, the number of beads and the initial length of the molecule. $A_{mol}$ is the cross-sectional area of the molecule and is calculated based on a molecule diameter $D_{fibril}$ = 16.52 Å.

#### 2.1.2. Stage 2: Molecule Stretching before the Hyperelastic Limit

After unfolding, the molecule starts to stretch. At a low enough strain rate, the stretching of the molecule remains uniform until the distance between beads reaches the hyperplastic critical distance $r_{1}$. The stress and strain at the end of this stage are denoted as $\varepsilon_{2}^{M}$ and $\sigma_{2}^{M}$. They are given by:

(9) $$\{\begin{array}{c} \varepsilon_{2}^{M}=\frac{n\times r_{1}}{L_{0}}-1\;\sim \;31.77\%\\ F_{2}^{M}=K_{T0}\left(r_{1}-r_{0}\right)\sim =5\times 10^{-9}\;N\\ \sigma_{2}^{M}=\sigma_{1}+\frac{F_{2}}{A_{mol}}\sim 2.34\;\mathrm{GPa} \end{array}$$

The Young modulus in this stage is denoted as $E_{2}^{M}$ and can be calculated as:

(10) $$E_{2}^{M}=\frac{\Delta \sigma }{\Delta \varepsilon }=\frac{K_{T0}\times L_{0}}{nA_{mol}}\;\sim 7.67\;\mathrm{Gpa}$$

#### 2.1.3. Stage 3: Molecule Stretching after the Hyperelastic Limit

Once the distance between beads reaches the hyperelastic critical distance $r_{1}$, the stress on the molecules starts to increase more rapidly (since $K_{T1}$ > $K_{T0}$) until the distance between beads reaches the breaking distance $r_{b}$, after which the bonds break, and the stress drops to zero. The stress and strain at the end of this stage are denoted as $\varepsilon_{3}^{M}$ and $\sigma_{3}^{M}$. They are given by:

(11) $$\{\begin{array}{c} {\;\varepsilon }_{3}^{M}=\frac{n\times r_{b}}{L_{0}}-1\;\sim \;52.04\%\\ F_{3}^{M}=K_{T1}\left(r_{b}-\bar{r_{1}}\right)\sim 2.40\times 10^{-8}\;N\\ \sigma_{3}^{M}=\sigma_{1}+\frac{F_{3}^{M}}{A_{mol}}\sim 11.21\;\mathrm{Gpa} \end{array}$$

The Young modulus in this stage can therefore be calculated as

(12) $$E_{3}^{M}=\frac{\Delta \sigma }{\Delta \varepsilon }=\frac{K_{T1}\times L_{0}}{n\times A_{mol}}\;\sim \;43.72\;\mathrm{Gpa}$$

Finally, the stress–strain for the tropocollagen molecule is therefore given by:

(13) $$\sigma^{M}=\left\{\begin{array}{ccc} E_{1}^{M}\times \varepsilon & 0<\varepsilon <\varepsilon_{1}^{M} & \left[0<\varepsilon <1.36\%\right]\\ \sigma_{1}^{M}+E_{2}^{M}\left(\varepsilon -\varepsilon_{1}^{M}\right) & \varepsilon_{1}^{M}<\varepsilon <\varepsilon_{2}^{M} & \left[1.36\%<\varepsilon <31.77\%\right]\\ \sigma_{2}^{M}+E_{3}^{M}\left(\varepsilon -\varepsilon_{2}^{M}\right) & \varepsilon_{2}^{M}<\varepsilon <\varepsilon_{3}^{M} & \left[31.77\%<\varepsilon <52.04\%\right]\\ 0 & \varepsilon_{3}^{M}<\varepsilon & \left[52.04\%<\varepsilon \right] \end{array}\right.$$

### 2.2. Stress–Strain Curve for Collagen Fibril with No Crosslinks

**Figure 2:**
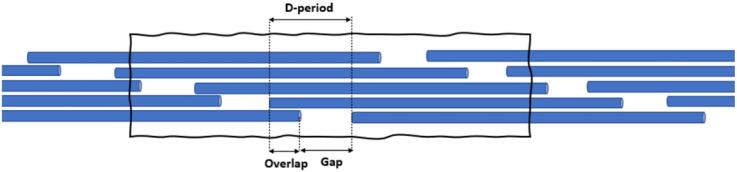
Collagen fibril Gap/Overlap structure.

The collagen fibril is constructed by replicating tropocollagen molecules in the radial direction orthogonally to their principal axis to form a cylindrical shape. The packing of the molecules is found to be in the form of a quasi-hexagonal array, where each group of five molecules packs together to form a microfibril [33]. Within the microfibril, molecules exhibit 5 gap/overlap regions along the fibril’s length. The collagen has an average periodicity $D_{period}\sim \;67\mathrm{nm}$ (1 gap + 1 overlap), as illustrated in Figure 2. The ratio of gap to $D_{period}$ ranges from 0.54 to 0.60 [34,35]. In the present work, we assume that molecules are hexagonally packed and that the fibril diameter (noted ϕ) is variable.

The potential force field governing the fibril is the same force field governing the tropocollagen molecule, with an additional pairwise interatomic Lennard-Jones (LJ) interaction between beads from different molecules. The additional term is responsible for keeping the fibril together in the radial direction [30].

(14) $$E=E_{inter}+E_{bond}+E_{angle}$$

The additional interatomic potential and force are given by:

(15) $$\{\begin{array}{c} E_{inter}=4\varepsilon \left({\left(\frac{\sigma }{r}\right)}^{12}-{\left(\frac{\sigma }{r}\right)}^{6}\right)\\ F_{inter}=-24\varepsilon \left(\frac{2}{r}{\left(\frac{\sigma }{r}\right)}^{12}-\frac{1}{r}{\left(\frac{\sigma }{r}\right)}^{6}\right) \end{array}$$

where σ and ε represent, respectively, the characteristic distance and the minimum energy of the LJ forcefield. Parameters for the pairwise potential are given in Table 3.

#### 2.2.1. Stage 1: Unfolding of the Fibril

Similar to the single molecule, the first phase in a fibril tensile simulation is the unfolding stage. The maximum force in the bonds is identical to the single-molecule calculated force. Therefore, the stress and strain of the fibril at the end of this phase (noted $\varepsilon_{1}^{UF}$ and $\sigma_{1}^{UF}$) are given by:

(16) $$\{\begin{array}{c} \varepsilon_{1}^{UF}=\frac{n\times r_{0}}{L_{0}}-1\sim \;1.36\%\\ F_{1}^{UF}=-2K_{\theta }\;\left(\theta_{max}-\theta_{0}\right)\;\sim \;2.08\times 10^{-11}\;N\\ \sigma_{1}^{UF}=\frac{F_{1}^{UF}}{A_{mol}}\;\sim \;9.68\;\mathrm{Mpa} \end{array}$$

The Young modulus in this stage, denoted as $E_{1}^{UF}$, can therefore be calculated as:

(17) $$E_{1}^{UF}=\frac{\sigma_{1}^{UF}}{\varepsilon_{1}^{UF}}\;\sim \;711\;\mathrm{Mpa}$$

where the “*UF*” superscript indicates properties related to an uncrosslinked fibril.

#### 2.2.2. Stage 2: Stretching of the Fibril

**Figure 3:**
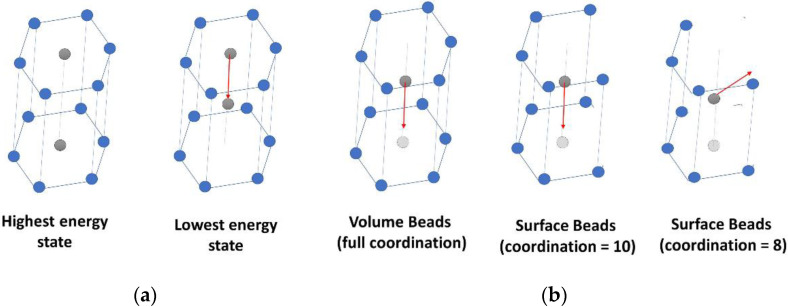
Beads’ displacement paths in the hexagonal collagen structure: (**a**) sliding bead position for lowest and highest energy states; (**b**) displacement paths for the sliding bead for the different coordination configurations.

Beyond unfolding, when a tensile force is applied to the fibril, molecules will start to stretch, and the beads will move in the axial direction. The motion of the beads will continue until they “slide” to the next potential well position. For this stage, we calculate the different properties by estimating the energy barrier the bead needs to cross before sliding. A possible approximation of the energy barrier is to assume that the atoms of the hexagonal lattice are fixed, except for the bead sliding, which moves only in the axial direction. The energy barrier can then be estimated as the difference between the lowest energy state where the bead is in between hexagonal layers and the highest energy state where the bead is at the same level as the hexagonal layers (Figure 3a). The value of these energies is calculated based on a summation of all LJ potentials of neighboring beads.

Numerical estimation of the barrier is calculated using a numerical MATLAB code and is found to be $\Delta E_{barrier}=6.29\;\mathrm{Kcal}/\mathrm{mol}$. This barrier is compensated during the stretching by the bond energy pulling the bead. The elongation of the bond and the corresponding force required to pull the bead out of the potential well are then given by:

(18) $$\{\begin{array}{c} \Delta r_{barrier}=\sqrt{2\frac{\Delta E_{barrier}}{K_{T0}}}\sim 0.86\;Å\\ F_{barrier}=K_{T0}\times \Delta r_{barrier}\sim 1.02\times 10^{-9}\;N \end{array}$$

During molecule sliding, when the bead starts moving along the axis, the “perfect” hexagonal structure is broken, and the beads slightly move out of position to minimize their energy and compensate for the moving bead. Therefore, the abovementioned assumption slightly underestimates the energy barrier. To overcome this problem, we use a molecular dynamics simulation to estimate the real energy barrier by moving the bead along the sliding direction and relaxing the rest of the beads at each step. The new equilibrium state is then obtained, and a more accurate energy barrier is found to be $\Delta E_{barrier}^{UF}=11.36\;\mathrm{Kcal}/\mathrm{mol}$. The use of MD to obtain the energy barrier in this problem does not defy the purpose of this work as only a few beads are simulated, which guarantees the accuracy of the estimation. We calculate the corresponding elongation and bond forces on the molecule using the new energy barrier.

(19) $$\{\begin{array}{c} \Delta r_{barrier}^{UF}=\sqrt{2\frac{\Delta E_{barrier}^{UF}}{K_{T0}}}\sim 1.15\;Å\\ F_{barrier}^{UF}=K_{T0}\times \Delta r_{barrier}^{UF}\;\sim \;1.37\times 10^{-9}\;N \end{array}$$

Once the bond forces reach $F_{barrier}^{UF}$, the bead will escape the potential well and jumps to the next favorable position, marking the onset of sliding. In terms of stress, only the molecules exhibiting the abovementioned behavior contribute to the fibril’s strength and should be considered. When bulk beads are in the potential well, they are coordinated to 12 beads (6 from each layer). If the molecule is on the surface, the coordination number can be 10, 8, 6, or 4. For atoms with 4, 6 or 8 neighbors (2, 3, or 4, respectively, from each layer), no energy landscape barrier must be crossed for the molecule to slide since these beads will not be constrained in the radial direction (Figure 3b). Therefore, they will not have any contribution to the overall strength of the fibril. For beads with ten neighbors (5 from each layer), the contribution to the strength of the fibril exists but is smaller than fully coordinated beads. Based on a built 3D model, we estimated that the molecules having beads with coordination numbers of 10 will range from 3% of the total number of beads (for fibrils 20 nm in diameter) to about 0.2% (for fibrils 500 nm in diameter). Adding the fact that the contribution of each of these molecules is already lower than that of bulk molecules, the effect of surface molecules can safely be neglected altogether.

An adjustment $C_{surf}$ coefficient can then be introduced to account for the molecules that are not contributing to the strength of the fibril. The adjustment coefficient is calculated as the ratio of bulk molecules to the total number of molecules and is given by:

(20) $$C_{surf}=\frac{A_{bulk}}{A_{fibr}}={\left(\frac{\phi -2D_{mol}}{\phi }\right)}^{2}$$

where $\phi {\;\mathrm{and}\;D}_{mol}$ are the diameter of the fibril and the diameter of the molecule, respectively. Numerically, $C_{surf}$ ranges from ~70% for 20 nm fibrils to ~99% for a 500 nm fibril.

**Figure 4:**
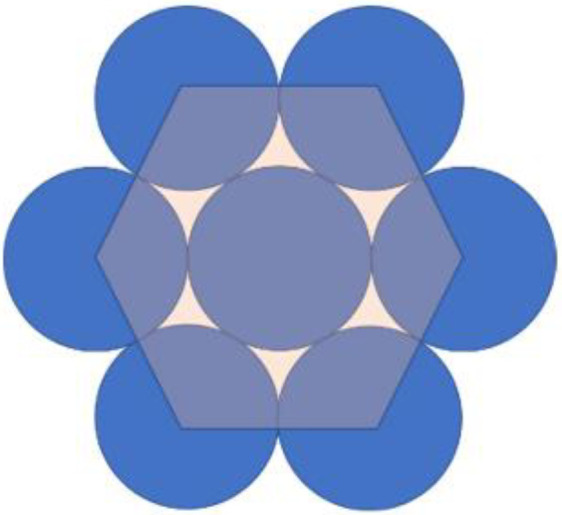
Collagen fibril packing coefficient description.

Since the area of the fibril is larger than the sum of the areas of the independent molecules (because of the hexagonal packing), we define the packing coefficient, denoted as $C_{Pack}$, as the ratio between the area covered by the molecules, denoted as $A_{molecules}$, and the total area of the hexagonal lattice, denoted as $A_{Hex}$ (Figure 4).

(21) $$\{\begin{array}{c} A_{molecules}=\frac{3\pi }{4}\times D^{2}\\ A_{Hex}=\frac{3\sqrt{3}}{2}\times D^{2}\\ C_{pack}=\frac{A_{molecules}}{A_{Hex}}=\frac{\pi \sqrt{3}}{6}\sim 90.7\% \end{array}\;$$

Another factor to be considered is the effect of the gap/overlap ratio. The total force in the cross-section is lower in the gap region since fewer molecules are present. However, the beads in that region will slightly move out of the axis to minimize their energies. Therefore, the cross-sectional area in the gap region will be reduced along with the total force, causing the stress to remain the same in both regions. The average stress caused by stretching is then given by:

(22) $$\sigma =\frac{F_{barrier}}{A_{mol}}\times C_{pack}\times C_{surf}$$

Therefore, the stress and strain at the end of this stretching phase are given by:

(23) $$\{\begin{array}{c} \varepsilon_{2}^{UF}=\frac{n(r_{0}+\Delta r_{barrier}^{UF})}{L_{0}}\sim \;9.70\%\\ \sigma_{2}^{UF}=\sigma_{1}^{UF}+C_{Pack}\times C_{surf}\times \frac{F_{barrier}^{UF}}{A_{mol}}\sim \;445\;\mathrm{Mpa} \end{array}$$

The Young modulus in this stage, denoted as $E_{2}^{UF}$, can therefore be calculated as:

(24) $$E_{2}^{UF}=\frac{\sigma_{2}^{UF}-\sigma_{1}^{UF}}{\varepsilon_{2}^{UF}-\varepsilon_{1}^{UF}}\;\sim \;5.24\;\mathrm{Gpa}$$

Since the stress depends on the diameter of the fibril ϕ, the presented numerical stress values are calculated based on a typical average fibril size (ϕ=100 nm).

#### 2.2.3. Stage 3: Sliding of Molecules

After the sliding starts, the forces on the bonds remain constant. Similar to the phase before, the stress on the fibril will not depend on the gap/overlap ratio. Therefore, the stress remains the same during this phase. Finally, the stress–strain for the un-crosslinked collagen fibril is therefore given by:

(25) $$\sigma =\left\{\begin{array}{ccc} \;E_{1}^{UF}\times \varepsilon & 0<\varepsilon <\varepsilon_{1}^{UF} & \left[\varepsilon <1.36K_{\theta }\right]\\ \sigma_{1}^{UF}+E_{2}^{UF}\left(\varepsilon -\varepsilon_{1}^{U}\right) & \varepsilon_{1}^{UF}<\varepsilon <\varepsilon_{2}^{UF} & \left[1.36\%<\varepsilon <9.70\%\right]\\ \sigma_{2}^{UF} & \varepsilon_{2}^{UF}<\varepsilon & \left[9.70\%<\varepsilon \right] \end{array}\right.$$

### 2.3. Stress–Strain Curve for a Fully Crosslinked Collagen Fibril

Enzymatic crosslinks are protein to protein bonds that make up most of the crosslinks in the collagen fibrils. They have been well quantified, understood, and modeled in several studies [36,37,38,39]. They are initially formed between a telopeptide (C or N molecule terminal) and a helical residue to produce an immature (divalent) crosslink connecting the end of the tropocollagen molecule to the nearest neighbor from an adjacent one. This immature crosslink may further react with another helical residue to produce a mature (trivalent) crosslink joining three collagen molecules by connecting the end of the molecule to two of the nearest neighbors from adjacent ones. Nonenzymatic crosslinks (or advanced glycation end products: AGEs), on the other hand, are products of a series of oxidative reactions that connect different amino acids to adjacent molecules. Their concentrations, however, are much smaller than enzymatic crosslinks, and they appear to correlate with numerous conditions such as diabetes and Alzheimer’s disease [40,41]. In this work, we only consider divalent enzymatic crosslinks.

For a fully crosslinked fibril, each of the terminals of the molecule—the carboxy-terminal telopeptide (noted C-terminal), and the amino-terminal telopeptide (noted N-terminal)—are connected to a randomly selected neighbor molecule via a hyperelastic bond defined in [30]. The properties of the crosslink fibril are given in Table 4.

When a crosslinking reaction occurs between molecules in the fibril, extra resistance to the sliding is created. The previously calculated energy barrier is no longer valid as the bead is now connected via an additional bond to one of its neighbors. Following the same methodology used to obtain the energy barrier for non-crosslinked molecules, we obtained using an MD simulation an energy barrier of $\Delta E_{barrier}^{CF}\sim 187\frac{\mathrm{Kcal}}{\mathrm{mol}}$. This energy barrier is about 16 times higher than the barrier with no crosslinks. Once again, the potential barrier will be crossed by the mean of the bond force pulling the molecule toward the tensile direction. Using the potential field expression, the elongation of the bead corresponding to the above-calculated barrier is given by:

(26) $$\Delta r_{barrier}^{CF}=\left(\bar{r_{1}}-r_{0}\right)+\sqrt{2\frac{\left(\Delta E_{barrier}^{CF}-C_{1}\right)}{K_{T1}}}\sim 4.59\;Å\;$$

The bond length when the energy barrier is crossed is therefore given by:

(27) $$r_{sliding}^{CF}=r_{0}+\Delta r_{barrier}\sim 18.59\;Å\;(>18.2\;Å)$$

The calculated bond length $r_{sliding}^{CF}$ is higher than the hyperplastic limit $r_{1}$. The molecule will, therefore, exhibit two stretching stages, and the mechanical behavior will then be as follows:

#### 2.3.1. Stage 1: Unfolding of the Fibril

At the unfolding stage, the behavior of the fully crosslinked fibril is identical to that of the non-crosslinked fibril. The stress and strain at the end of this stage (noted, respectively $\varepsilon_{1}^{CF}$ and $\sigma_{1}^{CF}$) are given by:

(28) $$\{\begin{array}{c} \varepsilon_{1}^{CF}=\frac{n\times r_{0}}{L_{0}}-1\sim \;1.36\%\\ F_{1}^{CF}=-2\;K_{\theta }\;\left(\theta_{max}-\theta_{0}\right)\;\sim \;2.08\times 10^{-11}\;N\\ \sigma_{1}^{CF}=\frac{F_{1}^{CF}}{A_{mol}}\;\sim \;9.68\;\mathrm{Mpa} \end{array}$$

The Young modulus in this stage, denoted as $E_{1}^{CF}$, can therefore be calculated as:

(29) $$E_{1}^{CF}=\frac{\sigma_{1}^{CF}}{\varepsilon_{1}^{CF}}\;\sim \;711\mathrm{Mpa}$$

where the “*CF*” superscript indicates properties related to a crosslinked fibril.

#### 2.3.2. Stage 2: Fibril Stretching before the Hyperelastic Limit

As the stretching starts, the bonds on each molecule will elongate until the interatomic distance reaches the critical distance $r_{1}$. For a fully crosslinked fibril, molecules on the surface are also connected to their nearest neighbors via divalent crosslinks, all molecules will then contribute to the strength of the fibril, and the $C_{Surf}$ coefficient will not be used. At the end of this stage, the stress and strain (noted, respectively $\varepsilon_{2}^{CF}$, $\sigma_{2}^{CF}$) are given by:

(30) $$\{\begin{array}{c} \varepsilon_{2}^{CF}=\frac{n\times r_{1}}{L_{0}}-1\sim \;31.77\%\\ F_{2}^{CF}=K_{T0}\left(r_{1}-r_{0}\right)\sim 5\times 10^{-9}\;N\\ \sigma_{2}^{CF}=\sigma_{1}^{CF}+C_{Pack}\;\frac{F_{2}^{CF}}{A_{mol}}\sim 2.115\;\mathrm{Gpa} \end{array}$$

The Young modulus in this stage, denoted as $E_{2}^{CF},$ can therefore be calculated as:

(31) $$E_{2}^{CF}=\frac{\sigma_{2}^{CF}-\sigma_{1}^{CF}}{\varepsilon_{2}^{CF}-\varepsilon_{1}^{CF}}\;\sim \;6.96\;\mathrm{Gpa}$$

#### 2.3.3. Stage 3: Fibril Stretching beyond the Hyperelastic Limit

Beyond the critical distance, the molecules will stiffen further, and the force on the bonds will increase more rapidly until the energy barrier for sliding is crossed at the bond length of $r_{3}=r_{sliding}^{CF}\sim 18.59\;Å$. The strain and stress at the end of this stage are denoted as $\varepsilon_{3}^{CF}$ and $\sigma_{3}^{CF}$, respectively, and are given by:

(32) $$\{\begin{array}{c} \varepsilon_{3}^{CF}=\frac{n\times r_{3}}{L_{0}}-1\sim 34.6\%\\ F_{3}^{CF}=K_{T1}\left(r_{3}-\bar{r_{1}}\right)\sim 7.67\times 10^{-9}\;N\\ \sigma_{3}^{CF}=\sigma_{1}^{CF}+C_{pack}\frac{F_{3}^{CF}}{A_{mol}}\sim 3.24\;\mathrm{Gpa} \end{array}$$

The Young modulus in this stage, denoted as $E_{3}^{CF}$, can therefore be calculated as:

(33) $$E_{3}^{CF}=\frac{\sigma_{3}^{CF}-\sigma_{2}^{CF}}{\varepsilon_{3}^{CF}-\varepsilon_{2}^{CF}}\;\sim \;39.65\;\mathrm{Gpa}$$

#### 2.3.4. Stage 4: Sliding of the Molecules

At a low enough strain rate, the crosslinks will break simultaneously, and the contribution of the crosslinks immediately cancels out. The force in the bonds will become higher than the critical force for sliding without the presence of crosslinks ($F_{barrier}^{UF}$), and the slipping of all molecules will initiate while keeping constant stress on the fibril equal to that calculated without the presence of crosslinks ($\sigma_{2}^{UF}$)

(34) $$\sigma =\left\{\begin{array}{ccc} E_{1}^{CF}\times \varepsilon & \;0<\varepsilon <\varepsilon_{1}^{CF} & \left[\varepsilon <1.36\%\right]\\ \sigma_{1}^{CF}+E_{2}^{C}\left(\varepsilon -\varepsilon_{1}^{C}\right) & \varepsilon_{1}^{CF}<\varepsilon <\varepsilon_{2}^{CF} & \left[1.36\%<\varepsilon <31.77\%\right]\\ \sigma_{2}^{CF}+E_{3}^{C}\left(\varepsilon -\varepsilon_{2}^{C}\right) & \varepsilon_{2}^{CF}<\varepsilon <\varepsilon_{3}^{CF} & \left[31.77\%<\varepsilon <34.60\%\right]\\ \sigma_{2}^{UF} & \varepsilon_{3}^{CF}<\varepsilon & \left[34.60\%<\varepsilon \right] \end{array}\right.$$

### 2.4. Stress–Strain Curve for a Partially Crosslinked Collagen Fibril

For a partially crosslinked fibril, the ratio of the crosslinks to the total number of molecule telopeptide terminals is denoted as β and is given by:

(35) $$\beta =\frac{n_{crosslinks}}{2\;n_{molecules}}\left(0\%<\beta <100\%\right)$$

where β=0% corresponds to the limit case of non-crosslinked fibrils, while β=100% corresponds to the case of fully crosslinked fibrils. For a partially crosslinked fibril, the total force in a cross-section equals the combination of forces on both types of molecules. Each molecule will either follow the behavior described for uncrosslinked fibrils or that of the fully crosslinked fibril, depending on whether crosslinks are restraining the sliding of that molecule. If the molecule is crosslinked via a single terminal, the crosslinking effect does not affect the molecule because of the gap/overlap structure of the fibril. Therefore, the only configuration at which the molecule will exhibit the crosslinked behavior is if both terminals are connected to neighbor molecules. The total stress of the fibril is then given by:

(36) $$\sigma =\alpha \times \sigma_{crosslinked}+\left(1-\alpha \right)\;\sigma_{un-crosslinked}$$

where α is the probability of a molecule being crosslinked with both the C and N terminals.

The number of molecules doubly crosslinked can be determined by analogy to a rigged tail coin flip problem, where the probability of having a tail on a coin flip is equivalent to the probability of each molecule terminal to be occupied by a crosslink (*β*). The probability of having both terminals of the molecule crosslinked is equivalent to having two tails on the coin flip problem and is therefore given by $\alpha =\beta^{2}$. Finally, the stress on a fibril with β crosslinks is given by:

(37) $$\sigma =\beta^{2}\times \sigma_{crosslinked}+\left(1-\beta^{2}\right)\;\sigma_{un-crosslinked}$$

(38) $$\sigma =\left\{\begin{array}{ccc} E_{1}^{C}\times \varepsilon & 0<\varepsilon <\varepsilon_{1}^{CF} & \left[\varepsilon <1.36\%\right]\\ \sigma_{1}^{C}+\left[\beta^{2}\times E_{2}^{CF}+\left(1-\beta^{2}\right)E_{2}^{UF}\right]\left(\varepsilon -\varepsilon_{1}^{C}\right) & \varepsilon_{1}^{C}<\varepsilon <\varepsilon_{2}^{UF} & \left[1.36\%<\varepsilon <9.70\%\right]\\ \left(\left(1-\beta^{2}\right)\sigma_{2}^{UF}+\beta^{2}\sigma_{1}^{CF}\right)+\left[\beta^{2}\times E_{2}^{CF}\right]\left(\varepsilon -\varepsilon_{1}^{CF}\right) & \varepsilon_{2}^{UF}<\varepsilon <\varepsilon_{2}^{CF} & \left[9.70\%<\varepsilon <31.77\%\right]\\ \left(\left(1-\beta^{2}\right)\sigma_{2}^{UF}+\beta^{2}\sigma_{2}^{CF}\right)+\left[\beta^{2}\times E_{3}^{CF}\right]\left(\varepsilon -\varepsilon_{2}^{CF}\right) & \varepsilon_{2}^{CF}<\varepsilon <\varepsilon_{3}^{CF} & \left[31.77\%<\varepsilon <34.60\%\right]\\ \sigma_{2}^{U} & \varepsilon_{3}^{CF}<\varepsilon & \left[34.60\%<\varepsilon \right] \end{array}\right.$$

## 3. Results and Discussion

**Figure 5:**
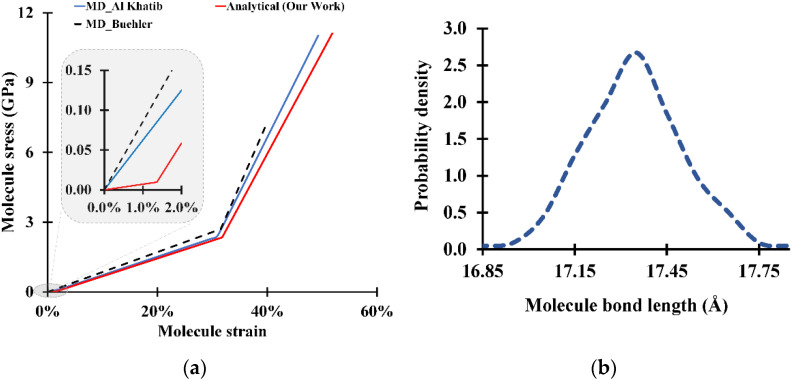
Tropocollagen molecule response to tensile load: (**a**) stress–strain curve for the tropocollagen molecules under tensile load (Al khatib et al., [29] and Buehler et al., [42]); (**b**) bond length distribution along tropocollagen molecule at a strain of 25%.

Stress–strain curves for the tropocollagen molecule are shown in Figure 5a. While our analytical formulation clearly shows the unfolding stage, all MD studies only show the second and third phases (stretching) and fail to detect the unfolding phase. However, beyond the onset of stretching, the results show a very strong agreement between the analytical calculations and the simulated molecular dynamics from previous studies [18,27]. In fact, for a single molecule, MD can perfectly mimic the molecule’s behavior using the coarse-grained method since the small number of particles allows MD to simulate the tensile test at a very slow strain rate [42].

In our mathematical approach, it is assumed that all bonds within the molecule behave similarly. Therefore, at a snapshot of a given strain, all bonds have the same length and the same bond energy. This assumption is not in full agreement with molecular dynamics. In fact, Figure 5b shows the bond length distribution along the molecule. Although the distribution is very narrow (standard deviation is ~0.15 Å), the bonds along the molecule have different lengths nonetheless. This distribution has very little effect on the overall stress because of the linearity of the bond force with respect to the strain: The overall stress will correspond to the stress induced by the average bond length regardless of how large the bond length variation is.

**Figure 6:**
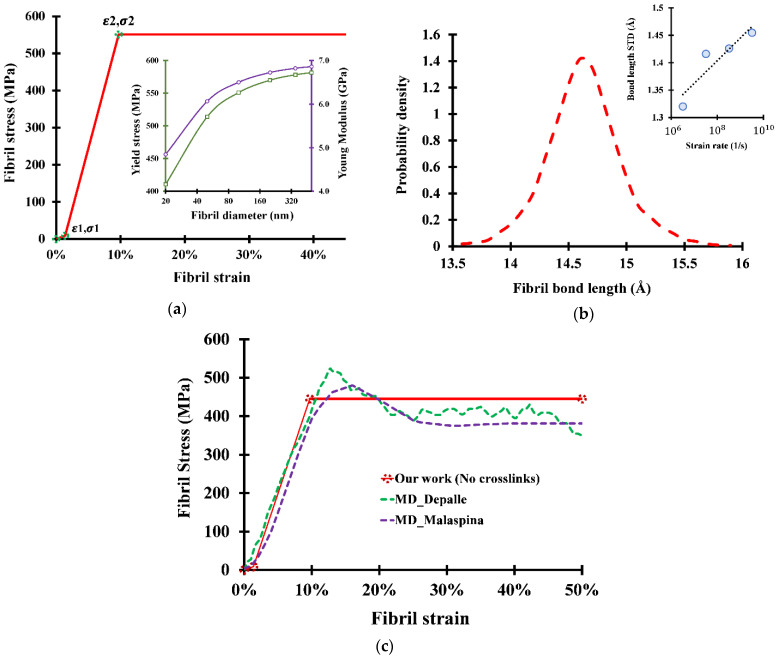
Non-crosslinked collagen fibril response to tensile load: (**a**) analytical stress–strain curve for a non-crosslinked collagen fibril with diameter *ϕ* = 100 nm; (**b**) bond length distribution for tropocollagen fibril (snapshot at a strain of 5.7%); (**c**) comparison of analytically derived stress–strain curve for a non-crosslinked collagen fibril with diameter *ϕ* = 25 nm and previous MD studies (Depalle et al. [18] and Malaspina et al. [27]).

At a fibrillar level, the obtained stress–strain curve for a non-crosslinked collagen fibril as well as the variation of the ultimate tensile stress and the Young modulus with respect to the fibril size are shown in Figure 6. The three described phases are clearly visible in the stress–strain curve, showing perfect plastic behavior. While the yield stress and the Young modulus increase with the fibril size (because the ratio of bulk molecules contributing to the strength increases with fibril diameter), the yield strain is found to be independent of the fibril size. For different fibril diameters (ranging from 20 nm to 500 nm), the ultimate tensile strength can range from 360 MPa to 580 MPa, while the Young modulus can range from 4.2 GPa to 6.86 GPa.

Figure 6c compares the analytical results with different molecular simulations and shows a good agreement with previously acquired results. For the comparison to be meaningful, the presented analytical curves are calculated based on a fibril diameter of 25 nm (typical fibril size used in MD studies). By comparing the two approaches, three main differences can be observed:

The first difference is that the calculated stress–strain curve shows no dissimilarity between the yield stress and the ultimate tensile stress, while MD simulations distinguish between properties. For example, Malaspina et al. [27] observed a yield stress of 420 MPa and an ultimate tensile stress of 480 MPa, while we calculated that both values are equal to 445 MPa for a 25 nm-diameter fibril.

The difference in behaviors emerges from the assumption that all bonds are stretched similarly and hence have the same energy. In fact, for MD simulations, the length of the bonds in the fibril shows a Gaussian distribution similar to the molecule bond length distribution (Figure 6b). Therefore, from an MD perspective, neither the bond lengths nor the bond energies are equal along the fibril. Some of the bonds will then reach the critical length for sliding while others are still trying to cross the sliding energy barrier. This phenomenon results in partial sliding, hence causing a gradual decrease in the Young modulus since the molecules already in the sliding phase will no longer be able to store additional energy and contribute to the stiffness, which will therefore mark the onset of the yield process. Once all bond lengths cross the critical sliding lengths, no additional energy can be stored in the bonds, and the perfectly plastic behavior starts.

Based on the variation of the standard deviation of the bond length distribution with respect to the strain rate (Figure 6b), we can observe a direct proportionality between the two variables, which suggests that the MD result might, at a low enough strain rate, converge with our calculated results. Although this claim cannot be verified since no MD tensile simulation can be reasonably slow, we can at least confirm that decreasing the strain rate narrows the gap between the yield and the ultimate stress, therefore altering the fibril behavior so that it is closer to the perfect plasticity.

The second difference is that a stress drop is observed in all MD studies directly after the ultimate tensile stress, while no stress drop is observed in our stress equations. We believe that there is no physical reason for the stress drop to occur. In fact, the reason behind this behavior is that once the sliding starts, not enough equilibration time is left for the molecules to rearrange before the sliding continues. This phenomenon will result in a higher cross-section, and therefore lower stress. The molecules will equilibrate the cross-sectional area at lower strain rates to compensate for the sliding, hence keeping the stress constant. This observation is in agreement with the difference in stress drops between different MD studies: for example, Malaspina et al. [27] observed about a 100 MPa drop (up to 30% strain), while Depalle et al. [18], using a strain rate ten times higher, observed a higher stress drop of about 130 MPa.

The third difference consists of the numerical values of the stresses. Although Figure 6c shows a good numerical agreement between the two approaches, it is important to note that the presented curve corresponds to a fibril size of 25 nm, which falls in the lower end of the fibril size spectrum. While type I collagen fibrils can reach up to 500 nm, the ultimate tensile stress can reach up to 580 MPa, and the Young modulus can reach up to 6.86 GPa (an increase of up to 30%). We can therefore conclude that MD slightly underestimates the strength of the collagen fibrils.

**Figure 7:**
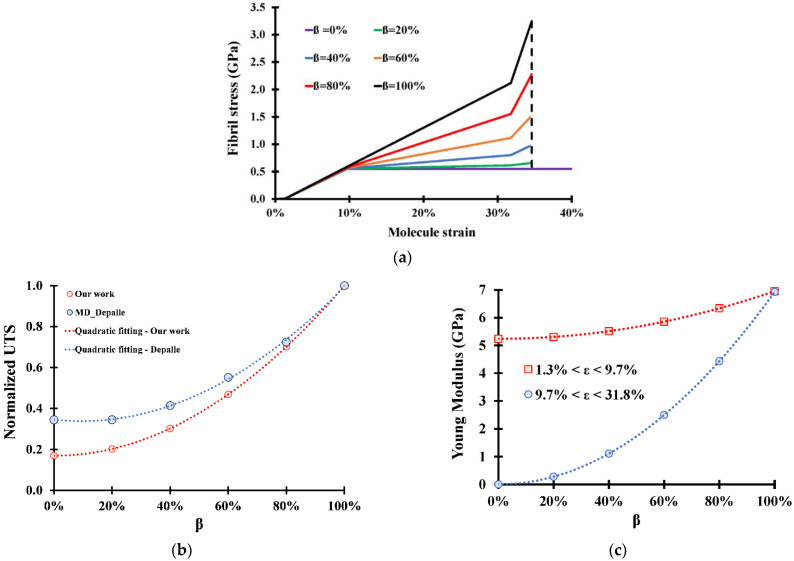
Partially crosslinked collagen fibril response to tensile load: (**a**) stress–strain curve for a partially crosslinked collagen fibril; (**b**) variation of the fibril UTS with crosslink density (Depalle et al., [18]); (**c**) variation of fibril stiffness with crosslink density (*ϕ* = 25 nm).

Adding enzymatic crosslinks to the fibril shows an increase in the overall stress for any strain value (Figure 7a). Comparing our analytical results to MD simulations for a partially crosslinked fibril yields three main observations:

The first observation is that our results do not show any correlation between the ultimate tensile strain and the crosslink concentration *β*, while molecular simulations [18,27,29] show that the ultimate tensile strain increases with increasing *β*. For example, Depalle et al. [18] observed an increase in ultimate tensile strain from 29% to 32%, with an increase in *β* from 60% to 100%. The interpretation behind this correlation lies in the mechanics of the stress drop observed in non-crosslinked fibrils. For MD simulations, the more molecules are crosslinked, the less molecules will be sliding for a given strain. The sliding molecules will exhibit the same stress drop seen in non-crosslinked fibrils, which will decrease the total stress on the fibril, causing the ultimate tensile stress and the ultimate tensile strain to decrease. This effect becomes less and less important with increasing *β*. It is important to note that even for *β* = 100%, the stress drop effect will still exist in MD since the bond lengths show a Gaussian distribution causing an early sliding of part of the molecules, and therefore causing the ultimate tensile strain to drop. Analytically, no matter how many molecule ends are crosslinked, the ultimate tensile strain will be aligned with the bond length corresponding to the energy barrier of the crosslinked molecules. The non-crosslinked molecules will be already sliding with constant stress without stress drop, thus not affecting the ultimate tensile strain. Therefore, we believe that molecular simulations underestimate both the ultimate tensile strength and the ultimate tensile strain.

The second observation is that both MD [18] and analytical approaches show a quadratic relationship between the Ultimate Tensile Strength and the crosslink density (Figure 7b). This dependence emerges from the fact that the two ends of the molecules must be crosslinked to exhibit the “fully crosslinked” behavior as explained in the methodology section.

The final observation is that although MD shows a slight increase in the Young modulus of the fibril, it fails to reveal the quadratic relationship it has with the crosslink density (Figure 7c). The reason behind this is those surface molecules do not contribute to the strength of the fibril if they are not crosslinked. The more crosslinks there are present, the fewer free surface molecules there are present, and the higher the Young modulus is. It is important to note that the dependency of the Young modulus on the crosslink density diminishes with the size of the fibril since large fibrils have an exceedingly small surface to bulk molecules ratio. The increase in Young modulus can reach up to 32% for fibrils of typical MD simulated (~25 nm), while it is only 1% for fibrils 500 nm in diameter.

**Figure 8:**
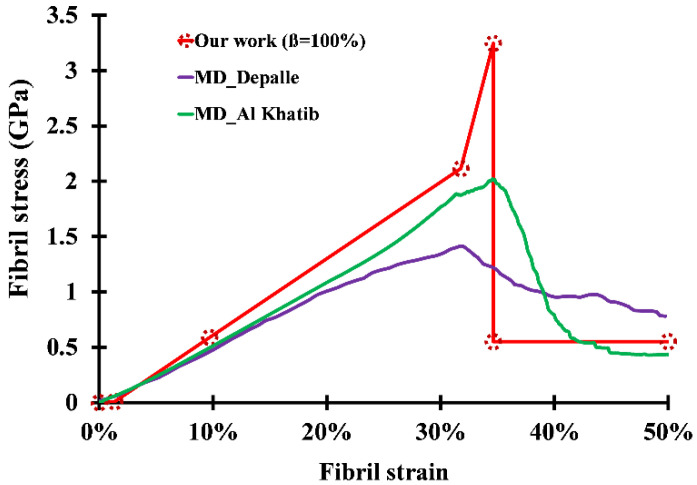
Comparison of analytically derived stress–strain curve for a fully crosslinked collagen fibril and previous MD studies (Depalle et al. [18] and Al khatib et al. [29]).

At the limit case where *β* = 100%, every molecule end is connected to a neighbor helical residue, and the fibril reaches its maximum stiffness (Figure 8). Neither the Young modulus nor the ultimate tensile strength depends on the fibril diameter, since all molecules now contribute to the stiffness of the fibril via their divalent crosslinks regardless of their surface/bulk positions. By comparing the results in Figure 8, it is clearly visible that the last stretching phase does show dissimilarities between the two approaches. We believe that the same partial sliding phenomenon due to the bond length distribution is the reason behind the quick stress drop once some of the molecules reach the sliding phase, therefore decreasing the ultimate tensile strength of the fibril.

Experimental data can hardly verify the presented results (or those obtained by molecular simulations) due to the very scarce available data and to the several chemical processes apart from the crosslinking (such as glycation, trivalent crosslinks, advanced glycation end products) involved in the actual type I collagenous tissues [36,43,44]. This excludes the possibility of obtaining tissue samples with the exact configuration discussed in this paper. However, our computed elastic modulus at different fibril mechanical behavioral stages is close to atomic spectroscopy experiments [7,45,46,47,48] where the reported range of values varies from 0.1 GPa to 11.5 GPa, while our calculated Young modulus is around 5 GPa, which is within this range, although towards the higher end. The potential reasons for the observed deviation between the analytical model and the measured experiments may be attributed to the abovementioned chemical processes not accounted for in our model or to limitations linked to the analytical method itself. The main limitation is that the methodology is built based on the coarse-grained model developed by Buehler [30]. Although the accuracy of his model has been proved, its empirical parameters emerge from MD simulations, which might impact the accuracy of the model. Moreover, our model, as well as previous MD studies, does consider all divalent crosslinks to be alike, which is not truly accurate, since several types of divalent crosslinks, with different concentrations and different bonding properties, are present in the collagen tissue (DHLNL, HLNL, PYD, DPD). Modeling the potential forcefields of each type of these crosslinks differently will require extensive molecular dynamics simulations, but will eliminate the abrupt nature of our analytical stress drop before the plastic zone seen in Figure 7a and Figure 8 since the crosslinks will not break at once, therefore attenuating the stress drop, which will prove very useful in improving the accuracy of the results. In addition, the assumption of constant stress along the fibril might be up for debate since several studies have proved that the Young modulus might differ in the gap and overlap regions [49,50]. These variations cannot be detected by our model since we assumed that the molecules on the gap region re-arrange to form a smaller cross-section and equate the stresses on the overlap zone. Load displacement curves (not in this work) can, on the other hand, be useful in providing additional information about the difference between the two zones [51]. Regardless of these limitations, we believe that this work can be considered a step toward the full characterization of collagen fibrils in live healthy human collagen tissues or those associated with specific diseases.

## 4. Conclusions

In this study, we used a different approach to characterize the mechanical behavior of type I collagen fibrils. The presented analytical results show similarities in the overall trend compared to previous molecular simulations. However, the different tensile phases showed a small deviation compared to MD, which we believe underestimates the Young modulus and the UTS of fibrils. This work can be further extended to access the tissue level length scales and characterize the collagen within the tissue simultaneously. Furthermore, researchers who do not have the resources to implement hierarchical fibrils models mimicking collagen I mechanical behavior using advanced MD simulations or numerical techniques are expected to use our analytical constructs.

## Figures and Tables

**Table 1:** Properties of tropocollagen molecule.

Parameter	Value
Molecule number of amino acids	3134
Molecule total mass [g/mol]	287,000
Number of beads per molecule	218
Mass of each bead [g/mol]	1316
Length along principal axis [Å]	3011

**Table 2:** Energy parameters of tropocollagen molecule.

Parameter	Value
$r_{0}$—Equilibrium distance [Å]	14.00
$r_{1}$—Critical hyperplastic distance [Å]	18.20
$r_{b}$—Bond breaking distance [Å]	21.00
$K_{T0}$—Stretching strength constant [Kcal/mol]	17.13
$K_{T1}$—Stretching strength constant [Kcal/mol]	97.66
$\theta_{0}$—Equilibrium bending angle [degree]	164–180
$K_{\theta }$—Equilibrium bending constant [Kcal/mol/rad^2^]	14.98

**Table 3:** Energy parameters of collagen fibril.

Parameter	Value
ε—Lennard-Jones energy [Kcal/mol]	6.87
σ—Lennard-Jones equilibrium distance [Å]	14.72

**Table 4:** Energy parameters of enzymatic crosslinks.

Parameter	Divalent Crosslinks	Trivalent Crosslinks
*r*_0_—Equilibrium distance [Å]	10.00	8.60
*r*_1_—Critical hyperplastic distance [Å]	12.00	12.20
*r_b_*—Bond breaking distance [Å]	14.68	14.89
*K_T*0*_*—Stretching strength constant [Kcal/mol]	0.20	0.20
*K_T*1*_*—Stretching strength constant [Kcal/mol]	41.84	54.60

## Data Availability

Not applicable.
